# DepthTools: an R package for a robust analysis of gene expression data

**DOI:** 10.1186/1471-2105-14-237

**Published:** 2013-07-25

**Authors:** Aurora Torrente, Sara López-Pintado, Juan Romo

**Affiliations:** 1 Functional Genomics Team, European Molecular Biology Laboratory, European Bioinformatics Institute (EMBL-EBI), Wellcome Trust Genome Campus, Hinxton, CB10 1SD, UK; 2 Departamento de Ciencia e Ingeniería de Materiales e Ingeniería Química, Universidad Carlos III de Madrid, Av Universidad, 30, 28911, Leganés, Spain; 3 Mailman School of Public Health, Columbia University, 722 West 168th Street, NY 10032, New York, USA; 4 Departamento de Economía, Métodos Cuantitativos e Historia Económica, Universidad Pablo de Olavide, Carretera de Utrera, Km 1, 41013, Sevilla, Spain; 5 Departamento de Estadística, Universidad Carlos III de Madrid, C/ Madrid, 126, 28903, Getafe, Spain

**Keywords:** Data depth, Robustness, R package, R commander plug-in

## Abstract

**Background:**

The use of DNA microarrays and oligonucleotide chips of high density in modern biomedical research provides complex, high dimensional data which have been proven to convey crucial information about gene expression levels and to play an important role in disease diagnosis. Therefore, there is a need for developing new, robust statistical techniques to analyze these data.

**Results:**

depthTools is an R package for a robust statistical analysis of gene expression data, based on an efficient implementation of a feasible notion of depth, the Modified Band Depth. This software includes several visualization and inference tools successfully applied to high dimensional gene expression data. A user-friendly interface is also provided via an R-commander plugin.

**Conclusion:**

We illustrate the utility of the depthTools package, that could be used, for instance, to achieve a better understanding of genome-level variation between tumors and to facilitate the development of personalized treatments.

## Background

The DNA microarrays and the oligonucleotide chips of high density are broadly used in modern biomedical research and in the study of numerous diseases like cancer or diabetes; also, in the last few years, the validity of this technology has become common in differentiation and development studies and in prenatal diagnostic testing for syndromes that involve small changes on chromosomes which are not seen through a microscope [1-6]. Microarrays allow gene expression profiling-based diagnosis and clinical risk stratification and facilitate the development of specific therapeutic treatments. They have made a great impact in the field of genomics, provide prognostic information for potential patients and play an important role in diagnostics and drug development. Additionally, a few recent studies have shown the application of this technique for revealing novel pathways or responses of important genes which were unstudied previously (see e.g. [7,8]).

Microarray gene expression data are complex and high dimensional (with usually small sample size) and suggest numerous statistical problems; to fully take advantage of the information conveyed by this technology and its impact in the understanding of living processes sound analyses of these data are needed. In this direction, a cohort of techniques, like for instance classifier algorithms, have been developed for gene expression data. A particularly intuitive, computationally inexpensive, and effective collection of methods suitable for the analysis of microarray data is the one proposed by [9]. This consists of a set of robust nonparametric tools, based on the concept of data depth, which generalizes unidimensional order statistics, ranks and medians to high dimensional data. A data depth notion measures the centrality of an observation within a sample and allows the definition of a natural ordering in a multidimensional space from center outwards and of other robust statistics such as trimmed means. Several depth definitions for multivariate data have been proposed and analyzed by [10-14], among others. However, most of these definitions of depth are intractable for dimensions larger than 3 or 4. The Modified Band Depth (MBD) proposed by [14] is computationally feasible for very high dimensions, what makes it specially appropriate for analyzing gene expression data. With this depth notion, it is possible, for instance, to define the most representative (or deepest) sample within a collection of observations which measure the expression (level) of a large set of genes in a group of individuals affected by a particular tumor type. This concept provides the basis for the statistical methods studied in [9]. In particular, classification techniques based on new similarity measures are proposed. The basic idea in these methods is to classify a new sample to the group having the representative (deepest sample) that is the most similar to the new observation.

More precisely, the nonparametric techniques described in [9] include: 1) a scale curve for measuring and visualizing the variability or dispersion of a set of tissue samples in a multidimensional (gene) space; 2) a rank test for deciding if two groups of samples come from the same population, e.g, for deciding whether they correspond to the same type of cancer or not; and 3) two classification techniques for assigning a new sample to one of *G* given groups (multi-class classification). This can translate into a more reliable diagnosis, based on sample profiles. These methods have been successfully applied to real microarray data and have been proven to be robust, efficient, and competitive with other procedures proposed in the literature, outperforming them in several situations [9].

The depthTools package implements these methods with an improved computational cost, allowing the visualization and analysis of gene expression data in a simple framework. Note that there are other packages implementing depth notions (such as the depth package), but they are not applicable for gene expression data, as they become computationally intractable for dimensions larger than 3 or 4. Thus, the depthTools package appears as a suitable choice to analyze gene expression data and should ultimately be useful for improving the characterization of tumor types, and for providing a clinical tool for early diagnosis of cancer and other diseases, or for abnormalities detection.

## Implementation

The statistical tools described in [9] and implemented in the depthTools package are based on the computation of the MBD of a high dimensional observation **y** within a collection **y**_1_,…,**y**_*n*_. The MBD of **y** with respect to **y**_1_,…,**y**_*n*_ represents the mean, over all possible pairs of distinct observations from **y**_1_,…,**y**_*n*_, of the proportion of coordinates of **y** that are between the corresponding components of two elements in the set **y**_1_,…,**y**_*n*_. The deepest sample has the largest of such average proportions.

In this section, we describe the functions implemented in the depthTools package, that also includes, for testing purposes, the prostate data, a subset of the data published by [15], normalized as described in the Prostate dataset subsection, and which contains both normal and tumor samples. The efficiency of the depthTools package stems from an alternative implementation of the MBD, described in the Methods subsection. Finally, in the R-commander support subsection, we describe briefly the implementation of a second package, the RcmdrPlugin.depthTools, that provides a user-friendly interface to make use of the depthTools package without the command line.

### Functions in the depthTools package

Function MBD:

The basic function in the package is MBD, which computes the depth of each element in a given data set and assigns a rank to it from center outwards. Additionally, this function allows the user to decide whether a plot of the data in parallel coordinates [16] will be returned. The most basic usage of the function is: MBD(x), where the mandatory argument x is an *n* × *d* data matrix containing the observations (samples) by rows and the variables (genes) by columns. In addition, several optional arguments can be provided. plotting is a logical value indicating whether the observations should be plotted (set to TRUE by default). In many situations, for instance in the context of classifying new data, the user will be interested only in knowing or envisaging the deepest sample of a group, which is, as mentioned before, the most representative gene expression profile within that group. For this reason, the default implementation of the MBD represents the dataset in a single colour, except for the deepest sample, which is distinctly drawn in a different one. In addition, it is also possible to depict each sample in grayscale, with intensities according to the order provided by the MBD, from deepest (light gray) to most external (dark gray).

Nevertheless, when the gene expression data set contains many samples, which are typically very irregular, such plots might become little informative or noisy, especially if the data set contains samples from different tissues or disease statuses. Therefore, an alternative is to picture the depth structure of the data, instead of by drawing all the curves, by plotting convex regions or bands, each containing a given proportion of the most central curves. To depict these bands in parallel coordinates, the minimum expression level of the samples that determine the band is computed for each gene, and the corresponding points are connected by straight lines, and analogously for the maximum expression levels. Representing these bands for different proportions helps understand how the data varies from center outwards. The logical parameters grayscale and band allow controlling, respectively, the use of gray intensities to reflect each sample position in the MBD ranking, and the representation of the bands. It is possible to draw different bands simultaneously through the argument band.limits. Further standard graphical parameters can be used to modify the aspect of the plot. Finally, we can pass to the parameter xRef an alternative data matrix containing a second collection of samples with respect to which the MBD is computed; this is useful when the user is interested in comparing the depth of a sample with respect to two different groups, as for instance in the rank test.

**Figure 1:**
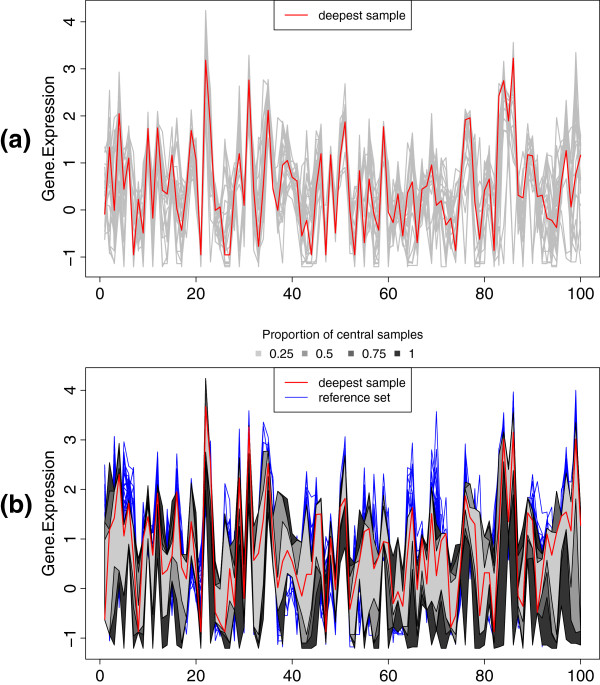
**MBD plots.** **(a)** Representation in parallel coordinates of 25 normal prostate samples, with the deepest one depicted in red. **(b)** MBD-based bands, for different proportions of central points (grayscale regions), corresponding to 25 normal prostate samples with respect to 25 cancer prostate samples (blue lines). The normal sample which lies the deepest in the reference collection of tumor ones is drawn in red.

To illustrate the use of the function MBD, we apply it to the data set prostate, included in the package. In particular, Figure 1 is obtained with the following code:

Panel (a) shows 25 normal samples, measured in 100 genes and represented in parallel coordinates, with the deepest gene expression profile depicted in red. Panel (b) corresponds to the computation of the MBD of these normal samples with respect to the 25 prostate cancer samples. The normal set is described in terms of the convex regions containing the percentages 25%, 50%, 75% and 100% of the most central curves, whereas the reference set is depicted in blue. The (normal) most representative sample with respect to the (cancer) reference set is shown by a red line. Note that in this case, the representative sample, that is, the one lying deepest in the prostate cancer set, is different from the most representative sample in the normal group, as one would expect.

In addition to possibly plotting the sample, the function returns a list containing two components: $ordering, a vector giving the ordering of the samples according to their depths, and $MBD, a vector with the computed depths.

**Figure 2:**
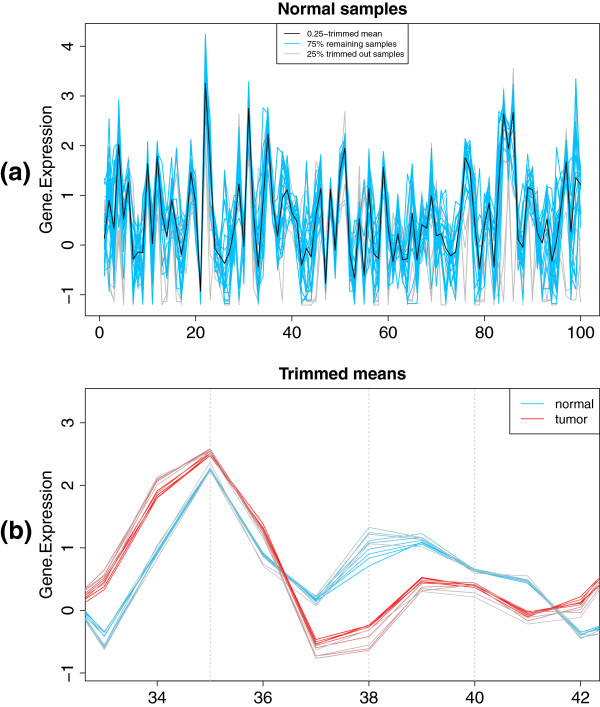
**Trimmed mean plots.** **(a)** Representation in parallel coordinates of the 0.25-trimmed mean of the normal prostate samples, in black. The trimmed out 25% most external points are depicted in gray; the remaining samples, used to compute the trimmed mean, are drawn in blue. **(b)** Trimmed means for different proportions of trimmed out points, corresponding to the normal (blue-gray) and cancer (red-gray) samples, for a subset of genes.

Function tmean:

When the data have been ordered from center outwards, it is possible to define a trimmed mean of such data with the function tmean, which computes the ordinary component-wise mean of the samples that remain after removing a pre-specified proportion of the most external curves. Though the main use of this function is internal, it is also possible to take advantage of it to get another visual representation of how the data varies from center outwards. A simple call to the function of the form tmean(x, alpha=0.2), where x is again an *n* × *d* data matrix and alpha is the proportion of observations that are trimmed out when computing the mean (0.2 by default), will provide an R list with two components: $tm.x, which is a matrix containing the deepest points of x after removing the proportion alpha of less deep samples, and $tm, a vector of length *d* with the alpha-trimmed mean (i.e., the ordinary mean of $tm.x). However, by setting the logical parameter plotting equal to TRUE, as in the following code:

we obtain a plot like that in Figure 2(a), where the 0.25-trimmed mean is visualized as a black line; additionally, the 0.25-trimmed sample, that is, the collection of samples remaining after removing the proportion 0.25 of less deep points are represented as blue lines, whereas the discarded samples appear as gray lines. These three colours can be modified with the parameter cols. Note that the samples removed in the plot are those defining the darkest region in Figure 1(b).

If instead of using a single value for the parameter alpha we choose a sequence of numbers in the range [0,1), we get a plot with the corresponding trimmed means, using a colour palette in which one extreme is chosen by the user, and corresponds to the curve closest to the ordinary mean (i.e., for the smallest value in alpha), whereas the other extreme is set to gray, and corresponds to the curve closest to the deepest sample (i.e., for the greatest value in alpha); this allows to envisage how the different genes vary their expressions from center outwards. Setting the logical parameter new to FALSE allows the comparison across different collections of samples, measured in the same set of genes. The following code:

computes the alpha-trimmed mean, for alpha in 0,0.1,…,0.9, for the normal and cancer samples separately, and leads to Figure 2(b), where we focus on a few genes (from the 33-rd to the 42-nd) to illustrate different situations. For instance, gene #35 shows very little variation across both normal (blue lines) and tumor (red lines) samples, and has similar expression values between both types. In contrast, gene #38 has considerably varying expression levels, that in addition depend on the disease status. Finally, gene #40 does not show a large variation across normal samples, but has a more volatile behaviour in cancer samples.

**Figure 3:**
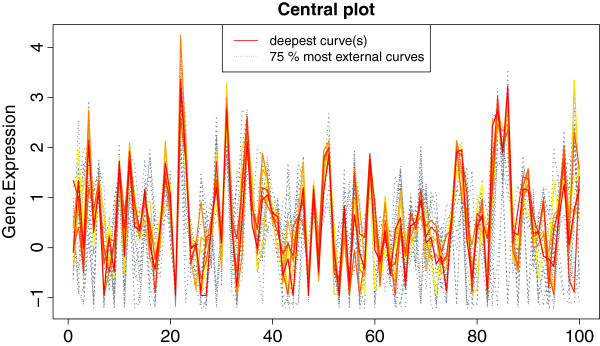
**Plot of the 25% most central normal prostate samples.** Representation in parallel coordinates of the normal samples in the prostate dataset with the 7 most central ones (25%) represented with solid lines and coloured from center outwards with a red-yellow palette; the remaining samples are shown as gray, dotted lines.

Function centralPlot:

This function provides alternative plots to the ones obtained with the function MBD. It allows focusing on a fixed percentage *p* of the most central curves (according to the MBD ordering), which is represented distinctly from the rest of samples, using different colours and/or line types (by default, red solid lines for the central curves and gray dashed lines for the external ones). Additionally it is also possible to incorporate to the plot the information about the depth ordering by setting the logical parameter gradient equal to TRUE and by defining a colour palette with the argument gradient.ramp, a vector with two components corresponding to the first and the last colours in the palette (red-yellow, by default). The first of such colors is used to draw the deepest sample. The following code:

leads to Figure 3, which shows the normal prostate samples, with the 25% deepest ones coloured from red to yellow, and the remaining 75% most external ones in gray. Note that this 25% of most central samples corresponds to the lightest region in Figure 1(b).

In addition, depthTools includes the following functions, which make use of the MBD and the tmean functions:

Function scalecurve:

Given the ordering defined by the function MBD, it is possible to determine, for each *p* ∈ [0,1], the band containing the proportion *p* of most central samples. The area of this band for each *p* defines the scale curve of the collection of samples, and the slope of the scale curve shows how the dispersion varies in the data.

The function scalecurve computes the scale curve of the *n* × *d* data matrix x and draws the corresponding plot by typing scalecurve(x). To allow the comparison of scale curves from different collections of samples (e.g. normal vs disease) there is an optional argument y, which takes as value a vector defining the class of each observation in x.

**Figure 4:**
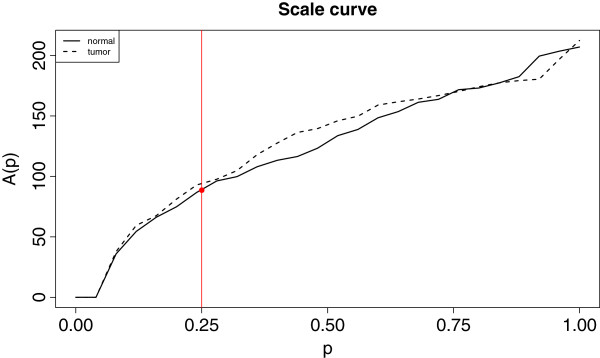
**Scale curves of normal and tumor prostate samples.** Scale curves for the normal and tumor samples included in the prostate data. The tumor samples (dashed line) have in general a larger dispersion than the normal ones (solid line). The red dot represents the spread of the 25% most central normal samples.

An application to the normal and tumor prostate samples is as follows:

As it can be seen in Figure 4, though the dispersion of both types of samples are similar, the tumor ones are more spread in general. In particular, the red dot in Figure 4 corresponds to the variability of the 25% deepest normal curves, and represents the area of the 0.25-band in Figure 1(b).

Function R.test:

In addition to visually comparing the dispersion of two collections of samples with the function scalecurve, it is also possible to decide whether two sets of samples come from the same population using the statistical rank test (see [9] for details), implemented in the function R.test. This allows the user, for instance, to decide if two groups of samples correspond to the same type of cancer or not. To use the function, we need to determine the four following arguments: an *n*_1_ × *d* data matrix x containing the observations from the first population, an *n*_2_ × *d* data matrix y with the observations from the second population, and two integers, n and m, representing, respectively, the size of the subsets randomly chosen from the first and second populations that will be used in the test. Additionally, due to this random component in the rank test, the user can initialize the random number generation with the optional parameter seed, which is set to 0 by default.

The following code, which uses random subsets of size 12:

yields a list with two components: the p-value of the rank test

and the value of the test statistic *W*.

As we can see, R.test rejects that the normal and tumor samples come from the same population (p-value < 0.01).

Functions classDS and classTAD:

These two functions implement the depth-based classification techniques DS and TAD, described in [9]. As any classification procedure, they both use a learning set, xl, in which the class of each sample, yl, is known, and a test set, xt, whose samples are to be classified into one of *G* known classes. In the DS procedure, which computes the trimmed mean of each group and classifies a new observation **y** from the test set in the class having the trimmed mean which is closest to **y**, we also need to specify the proportion alpha of observations that are trimmed out when computing this mean. In the TAD procedure, to determine the class of the new sample **y**, a weighted average distance from **y** to the most central points in each class is computed. The proportion alpha of most external points that are not considered in this weighted distance has to be specified as well. The output in both cases is a vector containing the class predicted for each observation in the test set.

As an example, we use the prostate data, considering the 20 first samples from each class as the learning set and the remaining samples as the test set:

Both methods classify correctly 9 out of 10 prostate samples. Notice that these techniques have been previously validated, and have been proven to be robust and very appropriate to analyze the complicated structures of gene expression data.

### Prostate dataset

This is a normalized subset of the real data published by [15]. The raw data comprise the expression of 52 tumor and 50 non-tumor prostate samples, obtained using the Affymetrix technology. The data were preprocessed by setting thresholds at 10 and 16000 units, excluding genes whose expression varied less than 5-fold relatively or less than 500 units absolutely between the sample, applying a base 10 logarithmic transformation, and standardizing each experiment to zero mean and unit variance across the genes. The 100 most variable genes were selected following the B/W criterion [17] and a random selection of 25 normal samples and 25 tumor samples was performed. The data are included in a matrix, where the 100 first columns correspond to the gene expression levels, and the last one contains the sample type: 0 for normal and 1 for tumor.

### Methods

The MBD for a *d*-dimensional point **y**_*i*_ = (*y*_*i*,1_,...,*y*_*i*,*d*_) in the sample **y**_1_,…,**y**_*n*_ is computed as

(1) MBD(yi)=n2−1∑1≤i1<i2≤nd−1×∑j=1dIminyi1,j,yi2,j≤yi,j≤maxyi1,j,yi2,j,

and represents the average proportion of coordinates of **y**_*i*_ inside the bands defined by every two different multidimensional points from the sample [14]. The implementation of this formula leads to nested for loops, which are known to be very inefficient in the R environment. Thus, to improve the computational cost of the MBD we used an alternative expression, developed in [18]. If we store the data in an *n* × *d* matrix **Y**, we can calculate the multiplicity of each value *y*_*i*,*k*_ in the corresponding *k*-th column of **Y**, rather than exhaustively search for all pairs of samples, and use this multiplicity to obtain the MBD of **y**_*i*_. It can be easily shown that this efficient expression can be computed by rewriting the MBD as follows. Given a data set *Y* = {**y**_1_,...,**y**_*n*_} and a point **y**_*i*_ ∈ *Y*, consider matrices

() $$Y=\;\left(\begin{array}{ccc} y_{1,1} & \cdots & y_{1,d}\\ \vdots & & \vdots \\ y_{n,1} & \ldots & y_{n,d} \end{array}\right)\;\text{and}\;\tilde{Y}=\;\left(\begin{array}{ccc} y_{(1),1} & \cdots & y_{(1),d}\\ \vdots & \; & \vdots \\ Y_{(n),1} & \cdots & Y_{(n),d} \end{array}\right),$$

where each column from **Y** has been increasingly ordered. Let *l*_*k*_ be the smallest index in the *k*-th column of $\tilde{Y}$ that verifies $y_{i,k}=y_{(l_{k}),k}$, and *η*_*k*_ be the multiplicity of *y*_*i*,*k*_ within the same column. Then, the MBD of **y**_*i*_ is given by

(2) MBD(yi)=1d×n2×∑k=1d(n−lk+1)(lk−1+ηk)−ηk2+ηk2,

assuming that ηk2=0 whenever *η*_*k*_ = 1.

To see this, note that

∑1≤i1<i2≤nImin(yi1,k,yi2,k)≤yi,k≤max(yi1,k,yi2,k)=∑1≤i1<i2≤nImin(yi1,k,yi2,k)≤yi,kImax(yi1,k,yi2,k)≥yi,k=∑1≤i1<i2≤nImin(yi1,k,yi2,k)<yi,k+Imin(yi1,k,yi2,k)=yi,k×Imax(yi1,k,yi2,k)>yi,k+Imax(yi1,k,yi2,k)=yi,k=(lk−1)·(n−(lk+ηk−1))+(lk−1)·ηk+ηk·(n−(lk+ηk−1))+ηk2=(n−lk+1)(lk−1+ηk)−ηk2+ηk2.

This is an alternative to the computation recently published by [19], in which they use a rank matrix to obtain the MBD; however, in formula (2), the cases in which there are variables (genes) with repeated values across different individuals (samples) are taken into account. Implementing expression (2) for MBD in R avoids the use of inefficient nested for loops, and reduces the computational time, for *n* data points in *d* dimension, from *O*(*d* × *n*^2^) (to compute the proportion for all pairs) to *O*(*n*^3/4^) (to compute the expression above), plus *O*(*d*) to reorder the data, using a variant of that of [20]. This is particularly important in the common situation where the number of dimensions (i.e. number of genes) is in the order of thousands.

An analogous expression can be obtained to compute the MBD of new observations **x** = (*x*_1_,...,*x*_*d*_), **x** ∉ **Y**, that is, the depth of sample **x** with respect to a data set which does not contain it. This is given by

MBD(x)=1d×n2×∑k=1d(n−l2,k)(l1,k+ηk)+l1,kηk+ηk2,

where *l*_1,*k*_ = |{*y*_(*i*),*k*_ < *x*_*k*_, 1 ≤ *i* ≤ *n*}| and *l*_2,*k*_ = |{*y*_(*i*),*k*_ ≤ *x*_*k*_, 1 ≤ *i* ≤ *n*}| represent the number of *k*-th coordinates in **Y** that are, respectively, smaller than *x*_*k*_ and not larger than *x*_*k*_, whereas *η*_*k*_ = *l*_2,*k*_−*l*_1,*k*_ provides the multiplicity of *x*_*k*_, assuming that ηk2=0 whenever *η*_*k*_ = 0, 1.

As a reference for the reduction in the computational time, we obtained the MBD of each of the samples contained in datasets of different sizes, using both implementations of this depth notion, with a computer with 2.53 GHz and 4 GB RAM; the average computational times, i.e., the total amount of time required to compute all the depths divided by the number of samples in the dataset, are displayed in Table 1 and show a remarkable improvement when the calculations are based on (2) rather than on (1).

**Table 1:** Computational cost of both MBD implementations

	**Computational cost**	
**Dataset size**	**Nested implementation. Time (s)**	**Matrix reordering. Time (s)**
10 × 500	0.1663	0.0151
25 × 500	1.1386	0.0148
10 × 1000	0.3570	0.0318
25 × 1000	2.2870	0.0277

Average time needed to compute the MBD of a sample of dimension 500 or 1000 with respect to a dataset with 10 or 25 samples (of the corresponding dimension), using the nested for loop implementation of the depth and the implementation based on reordering the columns of the dataset.

### R–commander support

**Figure 5:**
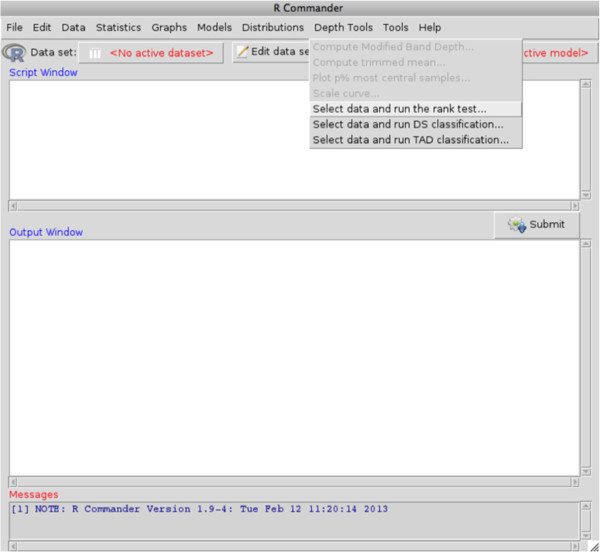
**Depth tools menu.** All the functions included in the depthTools package are available through the menu inserted in the R-commander bar.

The user has the option to add a new menu called “Depth tools" (see Figure 5) to the R-commander menu bar [21] by downloading and installing the RcmdrPlugin.depthTools package, available on CRAN. It provides an intuitive interface for the tools implemented in the depthTools package. This is specially useful for those researchers who prefer to avoid the command line code. The same functions described above are accessible from the menu Depth Tools, when the plugin is called. Each item in this menu bar opens a Dialog box with different buttons that allow tuning the parameters of the analysis to be performed.

**Figure 6:**
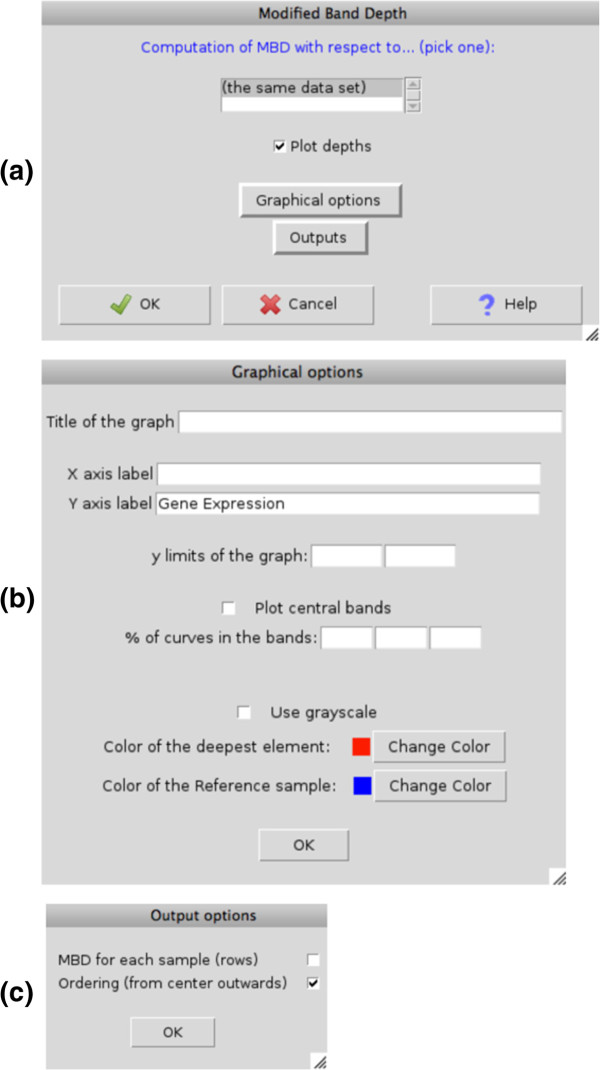
**MBD windows.** **(a)** Main window for the MBD computation in the R-commander. **(b)** Graphical window for adjusting the appearance of the MBD plot. **(c)** Output window for selecting which computations are stored as R objects.

As an example, we show the interface for the computation of MBD (see Figure 6(a)). Once the R-commander active dataset has been chosen, if we select ‘Compute Modified Band Depth...’ in the menu bar (Figure 5), the main window pops up and allows deciding whether the depth is computed with respect to the given sample or with respect to a different one. The user can also obtain the plot produced by the function MBD, and adjust its appearance with the Graphical options button (see Figure 6(b)). The outputs of the MBD computations are the depth and the order position from center outwards of each point, and can be selected to be stored as R objects (vectors) with the Outputs button (see Figure 6(c)).

## Discussion

We have developed depthTools, an R package that implements different robust statistical tools for the visualization and analysis of high dimensional gene expression data as illustrated with the prostate dataset. These tools make use of the MBD, a depth notion which is feasible for data with several hundreds or thousands of variables (genes), and of the ordering from center outwards that is derived from this depth. The computational cost of the MBD is drastically improved if we use an implementation based on reordering the columns of the data matrix rather than on exhaustively searching for all possible pairs of samples. An additional plugin for the R-commander has been designed to provide a friendly interface to users who are not familiar with the command line code. These methods can be easily applied to achieve a better understanding of genome-level variation between tumors and to facilitate the development of personalized treatments.

## Conclusions

The depthTools package implements the statistical tools based on the MBD in a very efficient way, greatly improving the computational cost of the original definition. It allows users to order from center outwards the samples in a high dimensional dataset like gene expression data, and to visualize the change in the dispersion through the scale curve or the central regions defined by different percentages of the most central samples. It is also possible to use the rank test to decide whether two sets of biological samples have the same disease status, and to identify the status of a new sample by means of the classification techniques DS and TAD. These tools will ultimately be useful for rapid diagnosis and efficient treatments. The R-commander plugin facilitates the use of the package avoiding the command line, and allows a wider use of these statistical methods.

## Availability and requirements

The package and the R-commander plugin have been developed for the statistical R environment ( http://www.R-project.org) and are freely available at http://cran.r-project.org/. The packages are accompanied by documentation files to facilitate their use.

**Project name:** depthTools

**Project home page:**http://cran.r-project.org/web/packages/depthTools/index.html and http://cran.r-project.org/web/packages/RcmdrPlugin.depthTools/index.html

**Operating system(s):** Platform independent.

**Programming language:** R platform.

**Other requirements:** No.

**License:** GPL (≥ 2)

**Any restrictions to use:** It is available for free download.

## Competing interests

The authors declare that they have no competing interests.

## Authors’ contributions

SLP and AT developed the algorithms and prepared the implementation of the depthTools and Rcmdr.depthTools packages. SLP and JR designed the statistical methods. All authors have read and approved the final version of the manuscript.
